# A Novel Adenovirus in Chinstrap Penguins (*Pygoscelis antarctica*) in Antarctica

**DOI:** 10.3390/v6052052

**Published:** 2014-05-07

**Authors:** Sook-Young Lee, Jeong-Hoon Kim, Yon Mi Park, Ok Sarah Shin, Hankyeom Kim, Han-Gu Choi, Jin-Won Song

**Affiliations:** 1 Department of Microbiology, College of Medicine, Institute for Viral Diseases, Korea University, Seoul 136-705, Korea; E-Mails: sylee163@gmail.com (S.-Y.L.); suerte8422@hotmail.com (Y.M.P.); 2 Division of Life Sciences, Korea Polar Research Institute, Incheon 406-840, Korea; E-Mails: jhkim94@kopri.re.kr (J.-H.K.); hchoi82@kopri.re.kr (H.-G.C.); 3 Department of Biomedical Science, College of Medicine, Korea University, Seoul 136-705, Korea; E-Mail: oshin@korea.ac.kr; 4 Department of Pathology, College of Medicine, Korea University, Guro Hospital, Seoul 152-703, Korea; E-Mail: sswords@naver.com

**Keywords:** adenovirus, *Siadenovirus*, Chinstrap penguin, Antarctica

## Abstract

Adenoviruses (family *Adenoviridae*) infect various organ systems and cause diseases in a wide range of host species. In this study, we examined multiple tissues from Chinstrap penguins (*Pygoscelis antarctica*), collected in Antarctica during 2009 and 2010, for the presence of novel adenoviruses by PCR. Analysis of a 855-bp region of the hexon gene of a newly identified adenovirus, designated Chinstrap penguin adenovirus 1 (CSPAdV-1), showed nucleotide (amino acid) sequence identity of 71.8% (65.5%) with South Polar skua 1 (SPSAdV-1), 71% (70%) with raptor adenovirus 1 (RAdV-1), 71.4% (67.6%) with turkey adenovirus 3 (TAdV-3) and 61% (61.6%) with frog adenovirus 1 (FrAdV-1). Based on the genetic and phylogenetic analyses, CSPAdV-1 was classified as a member of the genus, *Siadenovirus*. Virus isolation attempts from kidney homogenates in the MDTC-RP19 (ATCC® CRL-8135™) cell line were unsuccessful. In conclusion, this study provides the first evidence of new adenovirus species in Antarctic penguins.

## 1. Introduction

Adenoviruses are linear, double-stranded DNA viruses, with a genome ranging from 26 to 45-kbp and an icosahedral capsid [1]. Adenoviruses, which can infect the respiratory and gastrointestinal tracts, eyes and other organs, cause gastroenteritis and respiratory disease in many species [2]. The family, *Adenoviridae*, comprises five genera: *Mastadenovirus*, *Aviadenovirus*, *Atadenovirus*, *Siadenovirus* and *Ichtadenovirus* [3]. *Mastadenoviruses* infect a wide range of mammalian species, including man, monkey, dog, cattle, swine, mouse and bat [4,5,6,7,8,9,10]. *Aviadenovirus* have been identified in birds [11,12], and *Atadenovirus* have been isolated from reptiles, birds and mammals [13,14,15]. *Siadenovirus* has been detected in birds, frog and a tortoise [16,17,18,19], and *Ichtadenovirus* has been detected in fish [20]. 

Previously, adenoviruses had been isolated from various vertebrate species on all continents, except Antarctica. Recently, the South Polar skua adenovirus 1 (SPSAdV-1), the single known member of the species, *Skua siadenovirus A*, was discovered in dead South Polar skua (*Catharacta maccormicki*) collected near the King Sejong Station in Antarctica [21]. However, there are limited surveillance studies of adenovirus infection in Antarctic wild birds. The South Polar skua can be observed in Antarctica only during the breeding season and feed on the chicks of penguins. Moreover, the South Polar skua shares breeding grounds with the Chinstrap penguin (*Pygoscelis antarctica*), an endemic species [22]. To ascertain if Chinstrap penguins in Antarctica are also infected with adenoviruses, we conducted an exploratory study on samples collected during 2009 and 2010. 

## 2. Results and Discussion

Two of the 10 Chinstrap penguin carcasses (designated CSP09-1 and CSP09-2) were collected in the summer of 2009 and eight (CSP10-1 to CSP10-8) in early 2010. Fifty-six tissues, comprising lung, liver, kidney, heart, intestine, brain, colon, lymph node, spleen, trachea and wounded-bill, were tested. An approximately 1240-bp genomic fragment, including parts of two adjacent genes, that of pVI and hexon, was amplified from 28 tissues (lung, liver, kidney, heart, intestine and/or trachea) from eight Chinstrap penguins (CSP09-1, CSP10-1, CSP10-2, CSP10-3, CSP10-5, CSP10-6, CSP10-7 and CSP10-8), by nested PCR (Table 1). The Chinstrap penguin adenoviruses (CSPAdV-1) from eight Chinstrap penguins (from CSP09-1 to CSP10-8) were designated as CSPAdVno1 to CSPAdVno8, respectively. Adenovirus gene sequences, detected in PCR-positive tissues of individual Chinstrap penguins, were almost identical, suggesting widespread systemic infection.

**Figure 1:**
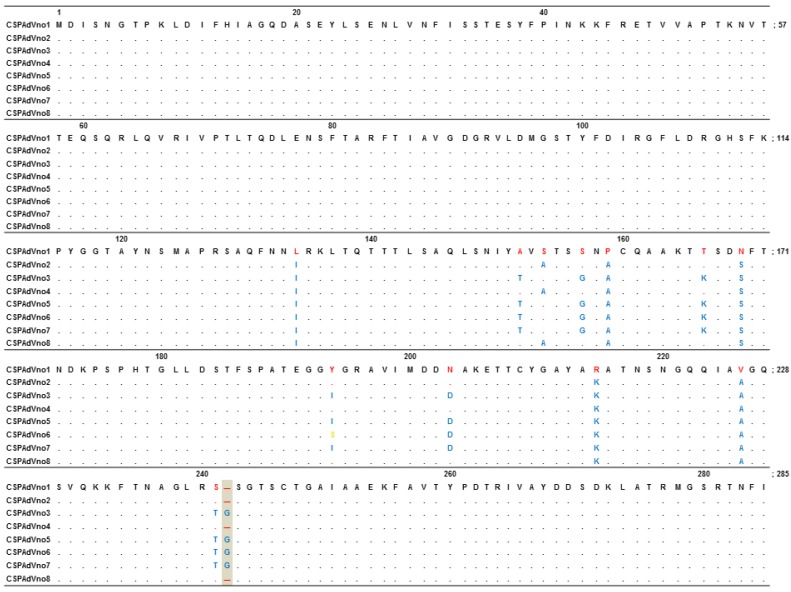
Differences of the amino acid sequence in the partial hexon gene of novel Chinstrap penguin adenoviruses. Within the 285-amino acid region, six amino acid changes were found between CSPAdVno1 and no2, no4 and no8 (residues 134, 154, 159, 169, 215 and 226), and 12-amino acid differences were found between CSPAdVno1 and no3, no5, no6 and no7 (residues 134, 152, 157, 159, 166, 169, 194, 203, 215, 226, 241 and 242). A deletion of an amino acid at position 242 was found in the hexon gene of CSPAdVno1, no2, no4 and no8 (gray rectangle, deletion of amino acid residue G; CAG, nucleotide sequences in 722–724 nt).

Pair-wise alignment by ClustalW showed a six-amino acid difference between CSPAdVno1 originating from a Chinstrap penguin collected in 2009 and CSPAdVno2, no4 and no8. Twelve amino acid differences were found between CSPAdVno1 and CSPAdVno3, no5, no6 and no7 (Figure 1). Particularly, CSPAdVno1, no2, no4 and CSPAdVno8 showed a deletion at position 242. As a result of sequence comparison with other types within the genus, *Siadenovirus*, the insertion of amino acid residue S at position 243 was found in the amino acid sequences of some CSPAdV-1 variants. Further study on the full genome sequence of CSPAdV-1 will be required to support the significance of sequence differences. 

**Table 1:** Detection of Chinstrap penguin adenovirus in various tissues by PCR, with GenBank accession numbers.

Samples No.	Tissues tested	Positives	Designation	Accession No.
CSP09-1	Lu, Li, K, Col, LN, Sp, Br	Lu	CSPAdVno1	KC593379
CSP09-2	W-B	-	-	-
CSP10-1	Lu, Li, K, Ht, Int, Tr	Lu, Li, K, Ht, Int	CSPAdVno2	KC593380
CSP10-2		Li, K	CSPAdVno3	KC593381
CSP10-3		Lu, Li, K, Int	CSPAdVno4	KC593382
CSP10-4		-	-	-
CSP10-5		Lu, K, Int, Tr	CSPAdVno5	KC593383
CPS10-6		Lu, Li, K, Ht, Int, Tr	CSPAdVno6	KC593384
CSP10-7		Ht, Tr	CSPAdVno7	KC593385
CSP10-8		Lu, K, Int, Tr	CSPAdVno8	KC593386

Abbreviations: Lu, lung; Li, liver; K, kidney; Ht, heart; Int, intestine; Tr, trachea; Col, colon; Sp, spleen; Br, brain; LN, lymph node; W-B, wounded-bill.

**Figure 2:**
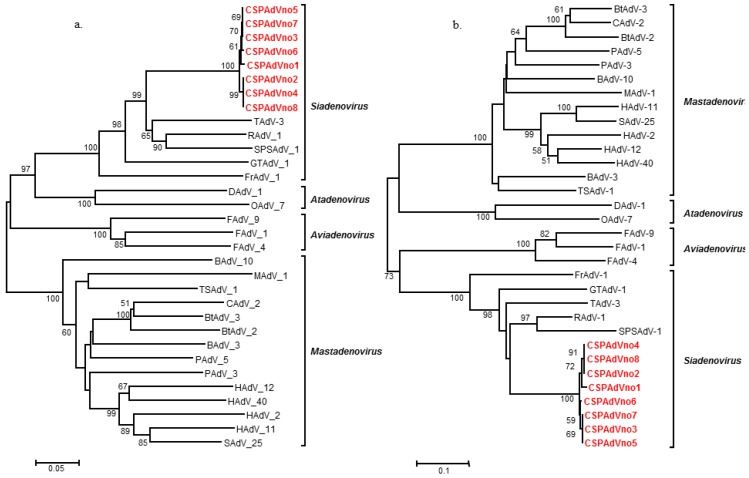
Phylogenetic relationship between Chinstrap penguin adenovirus (CSPAdV-1) and other adenoviruses. The phylogenetic tree, based on nucleotide (**a**) and amino acid sequences (**b**) of the hexon protein contained loop regions, was generated by the neighbor-joining method. CSPAdVno1 to no8 (KC593379 to KC593386) were compared with adenoviruses of five genera: bat adenovirus 3 (BtAdV-3, GU226970), canine adenovirus 2 (CAdV-2, U77082), bat adenovirus 2 (BtAdV-2. NC015932), porcine adenovirus 5 (PAdV-5, AF289262), porcine adenovirus 3 (PAdV-3, AB126117), bovine adenovirus 10 (BAdV-10, AAF82136), murine adenovirus 1 (MAdV-1, NC000942), human adenovirus 11 (HAdV-11, AY163756)), simian adenovirus 25 (SAdV-25, AF394196), human adenovirus 2 (HAdV-2, J01917), human adenovirus 12 (HAdV-12, X73487), human adenovirus 40 (HAdV-40, L19443), bovine adenovirus 3 (BAdV-3, AF030154), tree shrew adenovirus 1 (TSAdV-1, AF258784), duck adenovirus 1 (DAdV-1, Y09598), ovine adenovirus 7 (OAdV-7, U40839), fowl adenovirus 9 (FAdV-9, AF083975), fowl adenovirus 1 (FAdV-1, U46933), fowl adenovirus 4 (FAdV-4, NC015323), frog adenovirus 1 (FrAdV-1, AF224336), great tit adenovirus 1 (GTAdV-1, FJ849795), turkey adenovirus 3 (TAdV-3, AC000016), raptor adenovirus 1 (RAdV-1, EU715130) and South Polar skua adenovirus 1 (SPSAdV-1, HM585353). Scale bars indicate the number of nucleotide and amino acid substitutions per site. Bootstrap values are given at the respective nodes, as determined for 1000 iterations using the MEGA5.1 software [26].

Within the genus, *Siadenovirus*, CSPAdVno1 was shown to have a nucleotide (amino acid) identity of 71.8% (65.5%) with SPSAdV-1, 71% (70%) with raptor adenovirus 1 (RAdV-1), 71.4% (67.6%) with turkey adenovirus 3 (TAdV-3), 69.4% (66.5%) with great tit adenovirus 1 (GTAdV-1) and 61% (61.6%) with frog adenovirus 1 (FrAdV-1). The nucleotide and amino acid sequence identity of each strain of CSPAdV-1 was more than 97% and 96%, respectively (Table 2). However, the nucleotide and amino acid sequences of CSPAdVno3, no5 and no7 and CSPAdVno2, no4 and no8 were identical (Figure 1 and Figure 2). The nucleotide sequence identity of CSPAdV-1 was less (<33%) with other adenovirus genera, such as *Atadenovirus*, *Aviadenovirus* and *Mastadenovirus.* The G+C content of CSPAdVno1 and CSPAdVno2, no4 and no8 was found to be 37.44% and 37.2%, respectively. The partial hexon of CSPAdVno3, no5 and no7 had a G+C content of 37.42%.

**Table 2:** Nucleotide and amino acid sequence identity (%) between CSPAdVno1 and other CSPAdV-1 strains and representative *Siadenoviruses*.

Virus strain	Sequence identity (%)	
	nucleotide	amino acid
**CSPAdVno2**	**98.7**	**97.9**
**CSPAdVno3**	**97.5**	**96.1**
**CSPAdVno4**	**98.7**	**97.9**
**CSPAdVno5**	**97.5**	**96.1**
**CSPAdVno6**	**97.8**	**96.1**
**CSPAdVno7**	**97.5**	**96.1**
**CSPAdVno8**	**98.7**	**97.9**
SPSAdV-1	71.8	65.5
RAdV-1	71.0	70.0
TAdV-3	71.4	67.6
GTAdV-1	69.4	66.5
FrAdV-1	61.0	61.6

GenBank accession No.: CSPAdVno1 to CSPAdVno8 (KC593379 to KC593386), SPSAdV-1 (HM585353), RAdV-1 (EU715130), TAdV-3 (AC000016), GTAdV-1 (FJ849795), FrAdV-1 (AF224336).

For phylogenetic analysis, approximately 855-bp of the hexon gene, which contains structural loop regions that encode serotype-specific epitopes, were selected [23]. CSPAdV-1 showed the highest similarity with SPSAdV-1 and RAdV-1 and was classified into the genus, *Siadenovirus*, based on phylogenetic trees generated by the maximum-parsimony and neighbor-joining methods, implemented in MEGA5.1 (Molecular Evolutionary Genetics Analysis, 5.1) (Figure 2) and PAUP version 4.0b (Phylogenetic Analysis Using Parsimony, 4.0b) [24,25]. A novel adenovirus species is usually defined as one detected in a new host species and having more than a 5%–15% sequence difference at the nucleotide and amino acid levels compared with previously characterized adenovirus species [3]. Based on these criteria, we conclude that CSPAdV-1 (*Penguin siadenovirus A*) seems to merit the establishment of a new species for it. 

Isolation of CSPAdV-1 was attempted by inoculating the MDTC-RP19 (ATCC® CRL-8135™) cell line [27] with kidney homogenates, but all such attempts were unsuccessful. Failure of the isolation of RAdV-1 on the chicken embryonic liver cell or MDTC-RP19 cell line has been reported previously [28,29]. 

## 3. Experimental Section

### 3.1. Sample Collection

**Figure 3:**
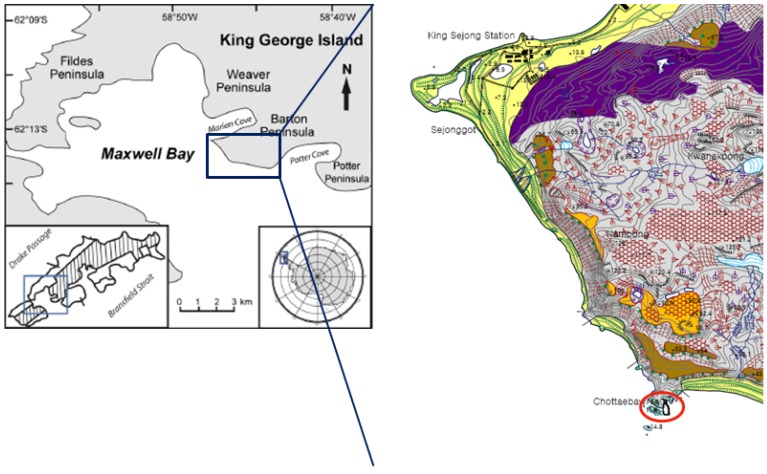
Collection site of Chinstrap penguin carcasses. Dead Chinstrap penguins were collected at Narębski Point (Antarctic Specially Protected Area; ASPA No. 171, red circle) on King George Island, Antarctica.

Chinstrap penguin carcasses were gathered at Narębski Point, located on the southeast coast of Barton Peninsula, King George Island, Antarctica (62°13'40''S–62°14'23''S and 58°45'25''W–58°47'00''W) during 2009 and early 2010 (Figure 3). All carcasses were identified, weighed and measured. Internal organs (lung, liver, kidney, heart, intestine, trachea, brain, lymph node and spleen) were dissected using sterile instruments and stored at −70 °C until use for adenovirus identification.

### 3.2. DNA Extraction and PCR

Total genomic DNA was extracted from tissue samples, using the High Pure PCR Template preparation kit (Roche, Indianapolis, IN), according to the manufacturer’s instructions. For screening of adenovirus infection, polymerase chain reaction (PCR) was used. PCR assay targeting capsid protein precursor pVI and the capsid protein hexon gene was performed using oligonucleotide primer pairs (outer: 5'-ACC (C/T)GG ATT AGC TGG TGA T-3', 5'-TAA TTT CTG TAT TCC TGT CCT-3'; inner: 5'-CCT GC(A/T) GAT CAA CTG GCT-3', 5'-GGA TCC CTA ACC ATT ATC GTA ATA-3'). The sequences of PCR primers were designated from the conserved region by the alignment of adenovirus sequences within the genus, *Siadenovirus*. PCR conditions were performed as follows: 1 cycle of 95 °C for 5 min followed by 14 cycles of denaturation at 95 °C for 40 s, one degree step-down each of 1 cycle annealing from 50 °C to 37 °C for 40 s, extension at 72 °C for 1 min, then 25 cycles of denaturation at 95 °C for 40 s, annealing at 42 °C for 40 s, extension at 72 °C for 1 min and, finally, at 72 °C for 5 min in a Mastercycler (Eppendorf, Germany).

### 3.3. Sequencing and Sequence Analysis

All PCR products were sequenced with the Big Dye terminator v3.1 cycle sequencing kit (ABI) and ABI3730 Automated DNA Sequencer (ABI). Nucleotide sequences were analyzed by ClustalW in MegAlign of DNAstar programs. Phylogenetic trees were generated by maximum-parsimony and neighbor-joining methods, implemented in MEGA5.1 and PAUP version 4.0b [24,25]. Topologies were evaluated by a bootstrap analysis of 1000 iterations by using MEGA5.1 software [26].

### 3.4. Isolation Attempts

For isolation attempts of CSPAdV-1, 5% (w/v) kidney homogenates of virus-infected penguins (CSP10-2 and CSP10-3) were inoculated onto MDTC-RP19 (ATCC® CRL-8135™), a lymphoblastoid cell line of turkey origin (*Meleagris gallopavo*) with high susceptibility to TAdV-3, a member of the genus, *Siadenovirus* [27]. Cultures were observed daily for the cytopathic effect, and the cells and supernatant were screened for the adenoviral hexon gene by PCR at each passage. 

## 4. Conclusions

Previous studies on sub-Antarctic and Antarctic penguins have shown that avian species in this region may become infected with various viruses, including paramyxoviruses, Newcastle disease virus, infectious bursal disease virus and influenza viruses [30,31,32,33,34,35,36,37,38]. The identification of a novel adenovirus species in Chinstrap penguins suggests the possibility of other viruses, including additional previously unrecognized adenoviruses in Antarctic birds. 

There is an increasing risk of infectious diseases being introduced into the Antarctic fauna, because of the increased numbers of people travelling to and within Antarctica [38,39]. Consequently, further studies in Antarctic birds may provide new insights into the emergence and dissemination of viral infectious diseases from Antarctica.
